# Evaluation of a Cost-Effective Ammonia Monitoring System for Continuous Real-Time Concentration Measurements in a Fattening Pig Barn

**DOI:** 10.3390/s19173669

**Published:** 2019-08-23

**Authors:** Shaojie Zhuang, Philippe Van Overbeke, Jürgen Vangeyte, Bart Sonck, Peter Demeyer

**Affiliations:** 1 Technology and Food Science Unit, Flanders Research Institute for Agriculture, Fisheries and Food (ILVO), Burg. van Gansberghelaan 115, 9820 Merelbeke, Belgium; 2 Animal Sciences Unit, Flanders Research Institute for Agriculture, Fisheries and Food (ILVO), Scheldeweg 68, 9090 Melle, Belgium; 3 Department of Animal Sciences and Aquatic Ecology, Faculty of Bioscience Engineering, Ghent University, Coupure links 653, 9000 Ghent, Belgium

**Keywords:** ammonia, tunable diode laser absorption, pig, field test, accuracy, emission

## Abstract

Ammonia (NH_3_) emission is one of the major environmental issues in livestock farming. Gas measurements are required to study the emission process, to establish emission factors, and to assess the efficiency of emission reduction techniques. However, the current methods for acquiring reference measurements of NH_3_ are either high in cost or labor intensive. In this study, a cost-effective ammonia monitoring system (AMS) was constructed from a commercially-available gas analyzing module based on tunable diode laser absorption (TDLA) spectroscopy. To cope with the negative measurement biases caused by differing inlet pressures, a set of correction equations was formulated. Field validation of the AMS on NH_3_ measurement was conducted in a fattening pig barn, where the system was compared to a Fourier-transform infrared (FTIR) spectroscopy analyzer. Under two test conditions in a fattening pig barn, the absolute error of the AMS measurements with respect to the average obtained values between the AMS and the FTIR was respectively 0.66 and 0.08 ppm_v_, corresponding to 5.9% and 0.5% relative error. Potential sources of the measurement uncertainties in both the AMS and FTIR were discussed. The test results demonstrated that the AMS was capable of performing high-quality measurement with sub-ppm accuracy, making it a promising cost-effective tool for establishing NH_3_ emission factors and studying NH_3_ emission processes in pig houses.

## 1. Introduction

Environmental pollution of ammonia (NH_3_) is one of the major issues in intensive livestock production [1]. Because of the strong impact on the environment and ecosystem, member countries of the European Union are obliged to either maintain or reduce the emission level according to targets set by the National Emission Ceilings Directive (2016/2284/EU). In Belgium and the Netherlands, newly-constructed pig barns are mandated to apply recommended emission reduction measures according to the Best Available Techniques (BAT) reference document [2], but information on the efficiency of the abatement techniques in practice remains limited. Early studies noticed that the reduction efficiency of the abatement measures might be inconsistent or paradoxical under certain conditions. For example, it was found that the removal efficiency of a chemical air scrubber varied significantly over time depending on the pH of washing water [3], and partially slatted floors in pig houses could lead to increased instead of decreased NH_3_ emissions if pigs excreted on solid floors [4,5]. Prolonged measurement is required to capture these situations and to obtain accurate information on the emission profile in an animal barn, but the reference methods are difficult to implement due to either high costs or high time requirements. For these reasons, in current practice, barn level emissions are assessed based on a simplified test protocol, which makes use of a minimum of six daily measurements to represent the yearly emission profile [6].

Wet chemistry is the most widely-accepted reference method for quantifying NH_3_ in animal houses [6,7,8,9]. In this method, air samples are continuously drawn through an acidic solution over a fixed period of time, and the amount of dissolved NH_3_ is thereafter analyzed in a laboratory. Theoretically, wet-chemistry-based methods are accurate and inexpensive, but their usefulness and efficiency are limited by several disadvantages in practice. Firstly, the methods only measure accumulative quantities, making them unsuited for monitoring temporal NH_3_ levels or the dynamic emission processes. Secondly, because the measurement procedure is complex and involves a considerable amount of manual handling, the accuracy of the method can be affected by human errors. Last but not least, as the measurement results are not immediately available, the method lacks practical convenience. Other reference methods for quantifying NH_3_ often rely on high-end gas analyzers. Examples of popular instruments include photoacoustic spectroscopy (PAS) analyzers [10,11,12], Fourier transform infrared spectroscopy (FTIR) analyzers [13,14,15], tunable diode laser absorption (TDLA) spectroscopy analyzers [16], and chemiluminescence analyzers [17,18]. The measuring processes of these systems are typically automated and thereby allow taking continuous real-time measurements at seconds- to minutes-level time resolutions. The manufacturer-claimed detection limits and accuracy range from ppm_v_–ppb_v_ levels depending on the gas sensing principle, system design, application condition, gas matrix, and calibration range. Nonetheless, not only are these analyzers costly, but also, most of them are not designed to perform measurement in harsh environments such as pig barns. The high-end instruments are therefore less commonly used for purposes other than research or small-scale measurement campaigns.

Electrochemical (EC) sensors and metal-oxide semiconductors (MOS) are low-cost sensors for gas measurement. Due to insufficient performance such as low accuracy, high drift and poor selectivity, these sensors are generally not accepted to provide reference measurements of NH_3_. Nonetheless, because of the affability of EC and MOS sensors, efforts have been put into overcoming the drawbacks in order to apply these devices for livestock applications. A portable monitoring system [19] and a subsequent upgrade [20] were developed for poultry facilities using off-the-shelf EC sensors. The reported accuracy of the upgrade version was ±3 ppm_v_ with a maximum 4.8 ppm_v_ drift over 48 h of continuous operation. To avoid signal drifts, the sensing components required at least 30 min of purging with fresh air after every independent measurement, but this approach restricted the time resolution of the system. A relatively new EC sensor model designed for long-term measurement under persistent exposure was tested in a cattle barn and a pig barn [21]. The sensor was shown to be sufficiently accurate above 1 ppm_v_ compared to a wet chemistry method. Nonetheless, it was unknown if and for how long the sensor could maintain its performance. A prototype of an MOS-based monitoring system was developed and tested in a poultry barn, where a 7% relative error compared to an EC sensor was reported [22]. As pointed out by the authors of [22], this MOS-based system might perform differently in pig barns due to cross-sensitivity with hydrogen sulphide and volatile organic compounds.

Optic-based NH_3_ sensing instruments are more capable of achieving high performance, but can be costly. A relatively more affordable optic-based device is the Axetris^®^ laser gas detection module for NH_3_. The market price of the device is approximately €6500, which is 5–20 times lower than the high-end instruments. An earlier evaluation in a laboratory condition showed that the measurement error of this device was less than 1 ppm_v_ across 0–68.8 ppm_v_ [23]. Another laboratory test demonstrated a reasonable accuracy at 0–25 ppm_v_, but at a higher range, the concentration was underestimated by up to 5% [24]. It was noticed that the analyzer output accuracy was strongly dependent on the gas pressure at the analyzer inlet [23], and recalibration or pressure compensation would be required if the pressure could not be maintained at the same level as the calibration condition.

The aim of this study was to construct a cost-effective ammonia monitoring system (AMS) for continuous real-time NH_3_ measurement in pig houses to establish emission factor, assess the efficiency of NH_3_ abatement techniques, and study emission processes. The AMS consisted of an Axetris^®^ laser gas detection module. To ensure a sufficient accuracy at variant inlet pressures, the pressure effect was investigated, and a set of equations was thereafter derived from the experimental data. Next, the gas analyzer module was integrated into a compact stand-alone system. Finally, the AMS was validated in a fattening pig barn and compared to a Fourier transform infrared spectroscopy (FTIR) gas analyzer in terms of the measurement accuracy on NH_3_ concentrations.

## 2. Material and Method

### 2.1. Gas Analyzer Module

The ammonia concentration measurement was carried out by an Axetris^®^ LGD F200-A NH_3_ original equipment manufacturer (OEM) module (Axetris AG, Kaegiswil, Switzerland), referred to as LGD F200. The device is based on tunable diode laser absorption (TDLA) spectroscopy, where the concentration of NH_3_ is estimated according to the Beer–Lambert law:

(1) $$\log\frac{I_{0}}{I}=\epsilon \cdot L\cdot c,$$

where:

- $I_{0}$ is the intensity of incident light in W·cm^2^;
- *I* is the intensity after transmitting through a medium, e.g., an air and gas mixture, in W·cm^2^;
- ϵ is an absorptivity coefficient of the absorbing species, e.g., ammonia, in mol^–1^·cm^3^·cm^–1^;
- *L* is the transmission distance through the medium in cm;
- *c* is the concentration of the absorption species in the medium in mol·cm^3^.

The light intensities before and after propagating through air samples were measured by the analyzer module. The absorptivity coefficient was determined for the target gas species at a given frequency and measurement condition. By choosing a representative absorption line of the target gas, which typically overlaps little with the absorption lines of other gas species, a high selectivity can be achieved with the TDLA technique [25]. The transmission distance was determined by the geometric architecture of the systems. The LGD F200 utilizes a flow-through closed-path design. A laser beam (linewidth ~0.05 nm) is emitted across a 20-cm measurement chamber and reflected once before reaching a photon detector, yielding a total 40-cm transmission distance inside the measurement chamber. The NH_3_ concentration is measured in real time while air samples are flowing through the measurement chamber.

The LGD F200 used in this study was factory calibrated for 0–100 ppm_v_ NH_3_ at 45% relative humidity under atmospheric pressure. The manufacturer-claimed accuracy was 2% full scale of the calibration, i.e., 2 ppm_v_. At the beginning of this study, the measurement chamber and the housing of the device were purged with dry nitrogen gas.

### 2.2. Laboratory Pressure Test

#### 2.2.1. Test Setup

**Figure 1:**
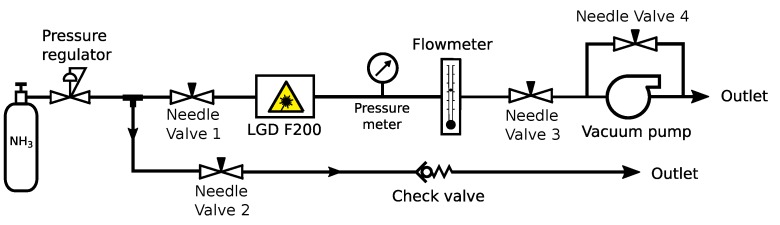
The laboratory pressure test setup of the Axetris LGD F200-A NH_3_ for measuring gases from cylinders under different inlet-pressures.

The setup shown in Figure 1 was used to test the LGD F200’s accuracy under different pressure levels using a premixed gas cylinder. The outlet pressure of the gas cylinder was reduced to a workable range with a stainless-steel pressure regulator. After the pressure regulator, the gas supply line was split into a measurement line and a bypass line with a T-shaped tube connector. Four needle valves (Model IQSDRV6, AirCompact NV, Gentbrugge, Belgium) were used to alter the flow path and regulate pressure level according to the configuration given in Table 1. During super-atmospheric pressure tests, the flow was driven by the outlet pressure of the gas cylinder. The magnitude of the pressure and flow rate were determined by the position of the pressure regulator and Needle Valve 3. In the meantime Needle Valve 2 was completely closed. During sub-atmospheric pressure tests, the flow was driven by both the cylinder outlet pressure and the suction force of a vacuum pump. The magnitude of the pressure and flow rate was adjusted by Needle Valve 1 and 4. In the meantime, Needle Valve 2 was fully opened to release the excess gas from the cylinder. The cylinder output pressure was fixed at a level slightly higher than the atmospheric pressure such that the flow rate from the gas cylinder was higher than the flow rate in the measurement line. All parts were connected by 4-mm inner diameter (I.D.) polytetrafluoroethylene (PTFE) tubes.

The inline pressure was measured with a 0–6 bar absolute piezo-resistive pressure transducer (Model PTI-S-AC5-12AS, Swagelok Belgium, Groot-Bijgaarden, Belgium), and the flow rate was measured with a 0–6 L_n_/min mass flow meter (Model F111AC, Bronkhorst, Ruurlo, The Netherlands). To avoid measurement bias due to adsorption, pressure measurement was taken at the downstream of the LGD F200, and the measured value was assumed representative for the pressure at the LGD F200 inlet. The LGD F200 data were obtained via the Axetris^®^ Graphical User Interface software (Axetris AG, Kaegiswil, Switzerland). The analogue outputs from the mass flow meter and the pressure transducer were logged by an Industruino IND.I/O microcontroller unit (ES Gear Ltd., Hong Kong, China).

#### 2.2.2. Experimental Pressure Test

Three certified gas cylinders (Air Liquide Benelux, Liege, Belgium) of respectively 5.03, 14.51, and 68.8 ppm_v_ NH_3_ mixed in N_2_ (±3% relative uncertainty) were used for testing the pressure effect on the accuracy of the LGD F200. Eight measurements were taken under conditions where the inline pressure was close to the atmospheric pressure. Additional measurements were taken under arbitrary pressure levels, ranging between 0.25 and 3 bar absolute. The sub-atmospheric condition contained 23 levels, and the super-atmospheric condition contained 11 levels. The 14.51-ppm_v_ cylinder was not used for testing super-atmospheric conditions due to depletion. The flow rate in the measurement line varied between 1.5 and 2.57 L_n_/min depending on the applied pressure. In the super-atmospheric pressure tests, the outgoing flow rate from the cylinder was set at least 0.5 L_n_/min higher than the flow in the measurement line. A leak test was performed each time after the measurement line was reconnected during the experiment. Absence of leakage was indicated by the flow rate converging to zero when the leak test configuration (Table 1) was applied. The LGD F200 was warmed up for one and a half hours to avoid potential systematic bias due to inconsistent instrument temperature. After adjusting the pressure to another level, the first 5 min of measurements were discarded, and then, an average NH_3_ concentration reading over 2 min was taken as the analyzer output. To compare the observed pressure dependency found on the LGD F200 with theoretical expectations, a simulation was carried out using the SpectraPlot online tool [26], which estimated the optic absorption spectrum of a given gas species according to the high-resolution transmission molecular absorption database 2012 (HITRAN2012) [27].

The absorption spectra were calculated for the 1.51-μm band for NH_3_ [28] under 0.3, 1, and 3 bar absolute. The NH_3_ concentration, temperature, and path length were set to 20 ppm_v_, 25 °C, and 40 cm, respectively.

#### 2.2.3. Formulation of the Compensation Equation

Candidate equations for approximating the mathematical dependency between the inline pressure at the LGD F200 and measurement bias were selected and adapted through trial-and-error. Two constraints were applied to the candidate equations: (1) the measurement bias equaled zero at the reference pressure condition, for which 1 bar absolute was used, and (2) the predicted gas concentration could not be negative at all pressure levels. The first constraint assumed that measurement biases, if any, at the reference pressure condition were exclusively caused by calibration errors. The second constraint was based on that negative absolute pressure does not physically exist for gases. The parameters of the equations were estimated via minimizing the residual sum of squares using a generalized simulated annealing algorithm [29]. The absolute and relative accuracy of the approximation was indicated by the root-mean-squared-error (RMSE) and relative root-mean-squared-error (rRMSE), which are calculated by:

(2) $$RMSE=\sqrt{\frac{1}{N}\sum_{i=1}^{N}{\left({\hat{C}}_{i}-C_{ref}\right)}^{2}},$$

(3) $$rRMSE=\sqrt{\frac{1}{N}\sum_{i=1}^{N}{\left(\frac{{\hat{C}}_{i}-C_{ref}}{C_{ref}}\right)}^{2}},$$

where $\hat{C}$ and $C_{ref}$ are respectively the estimated and reference concentrations.

### 2.3. System Setup for Field Application

**Figure 2:**
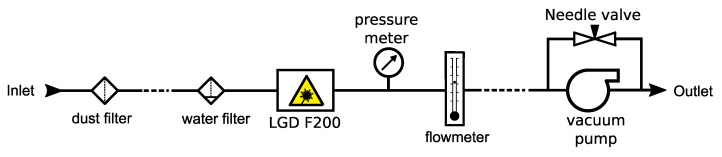
A generic measurement setup of the Axetris LGD F200-A NH_3_. The dashed lines indicate where the PTFE tube length was extended according to practical needs.

A generic measurement setup for the LGD F200 is shown in Figure 2. A particle filter was mounted at the inlet of the sampling tube, and a water filter was placed close to the inlet of the LGD F200. The tube length between the dust filter and the water filter was extended according to the distance between the analyzer and the measurement point. Pressure and flow rate measurements were taken after the LGD F200. A vacuum pump was used at the end of the sampling line to create an active flow. The in- and out-let of the pump was connected with a needle valve for controlling the vacuum strength. The pump, depending on the types, may need to be placed further away from the analyzer to avoid excessive vibration and electromagnetic interference, and the tube length between the pump and flow meter was adapted accordingly.

**Figure 3:**
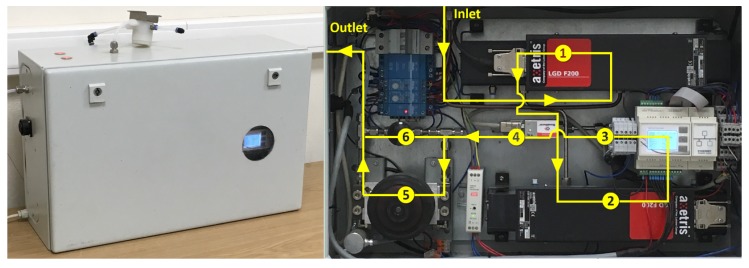
External (left) and internal (right) view of the AMS. The gas flow direction through the system is marked with arrows. The main components are indicated by circled numbers: ① Axetris LGD F200-A NH_3_, ② Axetris LGD F200-A CO_2_ (not tested in this study), ③ pressure transducer, ④ mass flow meter, ⑤ vacuum pump, ⑥ needle valve.

Based on the generic setup of the LGD F200 (Figure 2), a compact portable ammonia monitoring system (AMS) was assembled (enclosure size (L) 60 cm × (W) 38 cm × (H) 21 cm) (Figure 3). The parts in the gas measurement line were mostly connected with 4-mm I.D. stainless steel tubes and compression fittings, except the PTFE tube was used between the flow meter and the pump. The tube from the AMS inlet to the LGD F200 was coated with SilcoNert^®^2000 (SilcoTek GmbH, Bad Homburg, Germany) to reduce NH_3_ adsorption. Inline pressure and flow rate were measured with the same pressure transducer and mass flow meter as in the laboratory test, and the analogue output was logged with an Industruino IND.I/O microcontroller. Gas flow was driven by a vacuum pump with 6.8-L/min free-flow capacity (Model ML48.23-AW14, hyco Vakuumtechnik GmbH, Krailling, Germany). The NH_3_ measurements from the LGD F200 were logged with the Axetris^®^ Data Logger software. The AMS also consisted of an LGD F200-A CO2 OEM module, but the evaluation of the CO_2_ measurement was not in the scope of this study.

### 2.4. Field Experiments in a Pig Barn

#### 2.4.1. Test Facility

The field experiments were carried out in a pig barn at the Flanders research institute for agriculture, fisheries and food (ILVO ) (Merelbeke, Belgium). Gas measurements were made in one of the compartments with 8 pens and a service alley (floor area ~60 m^2^, volume ~240 m^3^) equipped with fully-slatted floors. The barn building utilized ground-channel ventilation. Beneath the slatted floor of the service alley was an air inlet channel connected to the outdoors. Water heating pipes were used to warm up the incoming air during cold seasons. An exhaust duct was installed on the compartment ceiling to create a passage between the compartment and an overhead exhaust channel. By reducing the pressure in the overhead exhaust channel, a suction force was created to draw outdoor air into the compartment via the ground channel. Ventilation rate was altered by adjusting the opening area of the exhaust duct with a diaphragm. Both the ventilation and inlet air heating were controlled by an automated climate control system based on the indoor temperature. Gas analyzing instruments were kept in an instrument room isolated from the pig rearing zone. The exhaust air from the gas analyzers was released to the outdoors. The temperature of the instrument room was maintained at 25.7 ± 1.9 °C with an air conditioner.

#### 2.4.2. Reference Ammonia Measurement

Reference measurements of NH_3_ concentration were provided by a Gasmet^™^ CX4000 Fourier-transform infrared (FTIR) spectroscopy analyzer (Gasmet Technologies Inc., Vantaa, Finland). The analyzer was calibrated for 0–150 ppm_v_ NH_3_ at 180 °C. The manufacture-claimed accuracy was a <2% calibration range, i.e., 3 ppm_v_. The measurement system was equipped with a Gasmet^™^ Multipoint Sampling System. During gas measurement, the sampled air sequentially flowed through the sampling system and the FTIR gas analyzer. The sampling tubes before the multi-sampler were heated to 180 °C with a 10-m heated hose. The measurement duration per update was set to 60 s, meaning that each measured value represented an average NH_3_ concentration over 60 s. During multichannel measurement, the measurement chamber was flushed with dry nitrogen for three minutes prior to starting a measurement in the next channel. No flushing was performed during single-channel measurement. Scheduled zero-calibration with dry nitrogen gas was performed once a day. The measured gas concentrations were logged by the Calmet^™^ v12.16 software (Gasmet Technologies Inc., Vantaa, Finland). Instrument inspection was performed within 5 months prior to this study by a professional from the distributor.

#### 2.4.3. Occupied Compartment Test

A three-week trial was carried out where forty-eight fattening pigs were reared in the compartment. The indoor temperature was maintained at 25.1 °C, varying between 24.2 and 26.1 °C, by the climate control system. The air exchange rate varied between 1056 and 2125 m^3^·h^–1^. Air samples were taken next to the exhaust duct in the compartment. Two 4-mm I.D. PTFE tubes of ~60 m long were used to transport the air samples respectively to the AMS and the FTIR. The last 10-m tube connected to the AMS inlet was exposed to the ambient temperature of the instrument room. The inlet nozzles of the sampling tubes were placed closely. The NH_3_ measurement interval of the AMS was approximately 1 s. In the first week, the inline pressure and flow rate were manually noted down, and from the second week onwards, continuous measurements with a 1-s update interval were taken. Because the FTIR was concurrently used for other tasks (unrelated to this study) via multi-channel sampling, the effective update interval was 48 min. A 2-micron stainless-steel particle filter (Model SS-6FW-MM-2, Swagelok Belgium, Groot-Bijgaarden, Belgium) and a polyvinylidene fluoride (Kynar^®^ PVDF) membrane filter (Genie^®^ 120 Hi-Flow, A+ Corporation, Gonzales, LA, USA) were used in the AMS sampling line. The particle filter was manually flushed with fresh air roughly every week to remove accumulated dust. The inlet of the FTIR sampling line was equipped with a 2-micron PTFE particle filter (Model SP53-T-2T, M&CTechGroup GmbH, Ratingen, Germany), but this filter was not purged or replaced during the test.

#### 2.4.4. Empty Compartment Test

A five-day trial was conducted in the compartment without the presence of pigs. The test started one day after the pigs were removed. Neither were the pens nor the slurry pit cleaned. Mechanical ventilation and heating were deactivated during the test period, but in-outdoor air exchange could still occur via natural air movement through the ground channel. Air samples were taken near the exhaust duct. A shared sampling tube was used to transport air from the compartment to the analyzers. A 2-micron stainless-steel particle filter was mounted at the inlet of the sampling tube. At the end of the sampling tube, a T-shaped tube connector was used to split the air stream for the AMS and the FTIR. The last 10-m tube connected to the T-shaped connector was exposed to the ambient temperature of the instrument room. The update interval of the AMS was approximately 1 s, and the update interval of the FTIR was 60 s.

#### 2.4.5. Data Processing and Analysis

The NH_3_ measurements from the AMS were firstly compensated for pressure based on the equation found in the pressure test. Next, A 60-s boxcar moving average was applied to the AMS data. Then, the AMS data were time-shifted based on the time-lag of the maximum cross-correlation against the FTIR data. Finally, the AMS data were resampled using linear interpolation according to the timestamps of the FTIR measurements. Data collected during the period when either of the analyzers was unavailable were discarded.

Bland–Altman analysis [30] was performed to assess the agreement between the AMS and the FTIR analyzer. Correlation between the instruments was indicated by the squares of Pearson’s correlation coefficient, i.e., R^2^. A total least squares (TLS) regression model was used to identify the homogeneous and the heterogeneous biases, such that $AMS=\beta_{0}+\beta_{1}\cdot FTIR$, where $\beta_{0}$ and $\beta_{1}$ were respectively the offset and span. Measurement error of the AMS was estimated with respect to the TLS prediction, and the accuracy was indicated by root-mean-squared-error (RMSE_TLS_) and relative root-mean-squared-error (rRMSE_TLS_), calculated according to Equations (2) and (3), respectively.

### 2.5. Data Processing

All data were processed and analyzed in R-3.5.2 software (R Foundation, Vienna, Austria) [31].

## 3. Results and Discussion

### 3.1. Laboratory Pressure Test

A nonlinear dependency of the LGD F200 output on inline pressure was observed (Figure 1a). At a pressure level around 1 bar absolute, the measurement bias was relatively small, which corresponded to the 0.8–1.1 bar absolute pressure range of the analyzer specification. Eight measurements were taken at reference conditions where the pressure levels ranged between 0.991 and 1.05 bar absolute. Assuming that the pressure related bias was negligible, these measurements yielded a linear calibration equation $NH_{3}^{ref}=-0.048+1.009\cdot NH_{3}^{meas.}$, where the RMSE and rRMSE were respectively 0.39 ppm_v_ and 1.1%. Outside this pressure range, the measured NH_3_ concentrations became noticeably underestimated, and the error grew larger as the pressure deviated further away from 1 bar absolute). At 0.25 and 3 bar absolute, the measured concentrations were underestimated by respectively 46% and 50%. Based on trial-and-error testing, $-a\cdot P^{b}+\left(1+a\right)\cdot P$ was found to be the best-suited formula for approximating the relative biases under sub-atmospheric pressure and ${\left(c\cdot {\left(P-1\right)}^{2}+1\right)}^{-1}$ under supper-atmospheric pressure, where *a*, *b*, and *c* are curve-fitting coefficients and *P* is the pressure. From the experimental data, the following set of equations was derived for compensating the errors at different pressure levels:

(4) $$\tilde{C}=\lambda \cdot C_{Rd},\;\mathrm{with}\;\lambda =\left\{\begin{array}{cc} {\left(-4.032\cdot P^{1.242}+\left(1+4.032\right)\cdot P\right)}^{-1} & \;\mathrm{if}\;P\leq 1\\ 0.248\cdot {\left(P-1\right)}^{2}+1 & \;\mathrm{if}\;P>1 \end{array}\right.,$$

where:

- $\tilde{C}$ is the estimated NH_3_ concentration after pressure compensation in ppm_v_;
- $C_{Rd}$ is the analyzer reading in ppm_v_;
- *P* is the inlet pressure in bar absolute;
- λ is a dimensionless correction coefficient.

**Figure 4:**
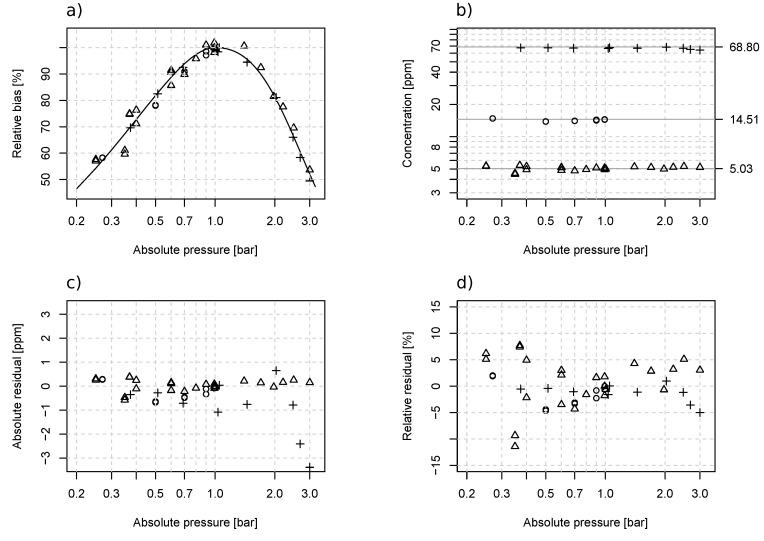
Pressure effect on the measurement bias of LGD F200 and correction results. The figures are respectively (**a**) the relative bias due to pressure and a fitted curve for compensation, (**b**) the concentration value after pressure compensation, (**c**) the absolute compensation residuals, and (**d**) the relative compensation residuals. Certified gas cylinders with 5.03 ± 0.15 (○), 14.51 ± 0.4 (△), and 68.8 ± 2.1 (+) ppm_v_ NH_3_ mixed in nitrogen were used in the test.

The measurement errors (Figure 4) were effectively reduced using Equation (4). The absolute and relative accuracy over the complete 0.25 to 3 bar absolute pressure range was respectively 0.47 ppm_v_ and 4.2%. For pressures below 0.8 bar absolute, the accuracy was 0.36 ppm_v_ and 5.1%, and for above 1.1 bar, the accuracy was 0.71 ppm_v_ and 4%. The RMSE of the sub- and super-atmospheric pressures was comparable to the 0.39-ppm_v_ accuracy found near 1 bar absolute, but the rRMSE was higher than the 1.1% error near 1 bar absolute.

**Figure 5:**
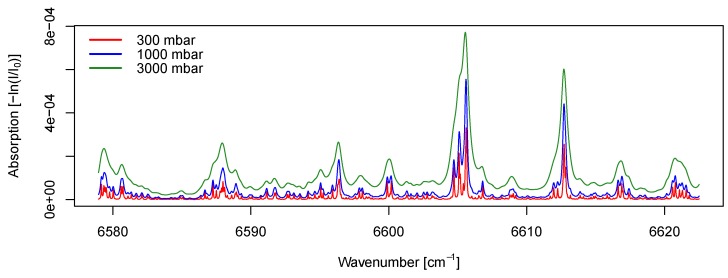
Simulation of optical absorption by 20 ppm_v_ of ammonia at 1.51 μm band under three pressure levels at 25 °C with a 40-cm transmission distance.

The Beer–Lambert law suggests that the amount of light absorption is directly associated with the number of gas molecules. When converting the number of substance molecules to ppm_v_, the free expansion of gases has to be accounted for according to the ideal gas law PV=nRT [25], where *n* is the number of substance molecules, *R* is the universal gas constant, *T* is the absolute temperature of the gas, *V* is the gas volume, and *P* is the pressure. The pressure level is also associated with the changes of the absorption line profile, known as pressure broadening [32]. At higher pressures, the spectral line shape of the absorption line will become wider. Although the spectral line shape also depends on natural broadening and Doppler broadening, the influence of these two mechanism was less evident above 0.13 bar absolute [32]. As a result of the pressure broadening, treating the absorptivity of a gas as a constant could lead to a biased estimation under different pressure levels. To illustrate the theoretical pressure effect on the optic absorptivity, a simulation was carried out at three pressure levels (Figure 5). The absorption of optic energy at the given frequencies was indicated by $-\log(I/I_{0})$ as defined by the Beer–Lambert law. It is clear that at higher pressure, the absorption was generally stronger and wider across the chosen frequency band. According to Equation (1), this should result in a higher estimated concentration. By default, the LGD F200 did not compensate for pressure. The changes in the absorption spectrum may explain the underestimation by LGD F200 under sub-atmospheric pressures. However, the underestimation under super-atmospheric pressures contradicted the simulation, and the reason was unclear.

### 3.2. Field Experiments in a Pig Barn

#### 3.2.1. Occupied Compartment Test

**Figure 6:**
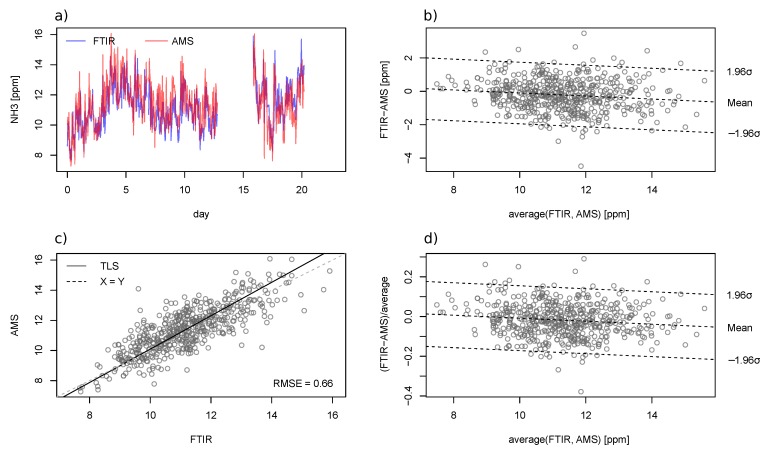
Comparison of sub-hourly NH_3_ measurements between AMS and FTIR in the occupied compartment test. The sub-plots are: the (**a**) time-series plot, (**b**) Bland–Altman plot of absolute differences, (**c**) scatter plot, and (**d**) Bland–Altman plot of the relative difference. The dashed lines in the Bland–Altman plots represent the regression line and 95% bound of residuals.

A good agreement between the AMS and the FTIR on the measured NH_3_ concentration was found (Figure 6). The RMSE_TLS_ and rRMSE_TLS_ of the AMS measurement were respectively 0.66 ppm_v_ and 5.9%, and the R^2^ was 0.64. In total, 511 valid NH_3_ concentration measurement pairs of sub-hourly measurements from the AMS and the FTIR were obtained over the three-week test period. The logging process of the FTIR was interrupted twice due to software malfunctioning, and respectively three and 1.5 days of measurements were lost from Day 13 and Day 21. The observed concentration varied between 7 and 17 ppm_v_. The middle 50% of the absolute difference between the AMS and FTIR ranged from −0.81–0.38 ppm_v_, and the relative difference ranged from −7.6%–3.4%. The TLS regression line was given by AMS=−0.884+1.099·FTIR. Both the absolute (Figure 6b) and relative difference (Figure 6d) between the two analyzers appeared to vary with the concentration levels.

**Figure 7:**
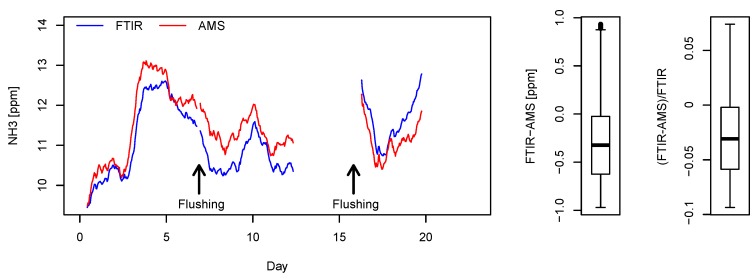
Twenty four-hour rolling average NH_3_ concentration measured by AMS and FTIR in the field test. Missing measurements were removed, and the remaining data were presented as continuous time series.

For measurements averaged over a 24-h window, the middle 50% of the absolute difference between the AMS and FTIR ranged from −0.62–−0.02 ppm_v_, and the relative difference ranged from −5.9%–−0.2% (Figure 7). The median of the absolute and the relative difference was respectively −0.32 ppm_v_ and −3.1%. The measured values by the AMS were higher than those of the FTIR between Day 0 and Day 13. Nonetheless, the FTIR measurements were higher than the AMS from Day 16 onwards despite that the setup remained the same throughout the test period.

**Figure 8:**
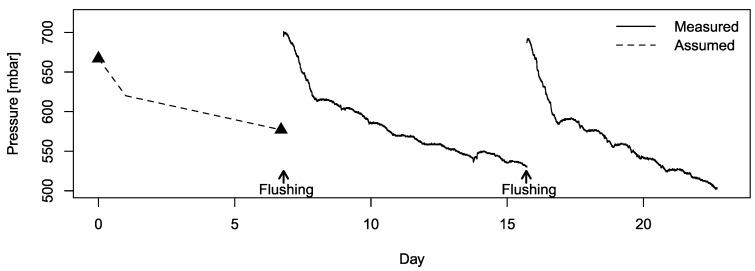
Inlet pressure change due to filter clogging over time. The starting and ending pressure levels (▴) in the first week were recorded manually.

Due to dust accumulation in the filter, the pressure in the AMS sampling line decreased significantly over time (Figure 8). Based on the continuous pressure measurement from the second week onwards, the most dramatic pressure decline, −3.3×10^−3^ and −4.2×10^−3^ bar absolute·h^−1^ on Day 7 and Day 16, respectively, occurred in the first day after the tube was flushed. From the second day after tube flushing, the decline rate slowed down to −0.5 × 10^−3^ and −0.65 × 10^−3^ bar absolute·h^−1^, respectively. The faster pressure decline at the beginning could be caused by a stronger suction force at the tube inlet, where dust particles of a relatively large size were drawn into the sampling tube to block the filter pores quickly. As the clogging led to weaker levels of suction, only small dust particles could be drawn into the tube, leading to a further drop in pressure. The pressure level also appeared to be more stable at a certain moment of the day, causing a ripple-like day-to-day pattern. This was likely associated with the daily refill of the feed bins and diurnal variation of the ventilation rate in the compartment. According to (4), the LGD F200 would underestimate the NH_3_ concentration by 12% at 0.6 bar absolute. To compensate for the pressure effect for the first seven days, a pressure drop profile was assumed (Figure 8), where a −2 × 10^−3^ bar absolute·h^−1^ and a −0.3 × 10^−3^ bar absolute·h^−1^ change rate were used respectively within and after the first 24 h of tube flushing.

#### 3.2.2. Empty Compartment Test

**Figure 9:**
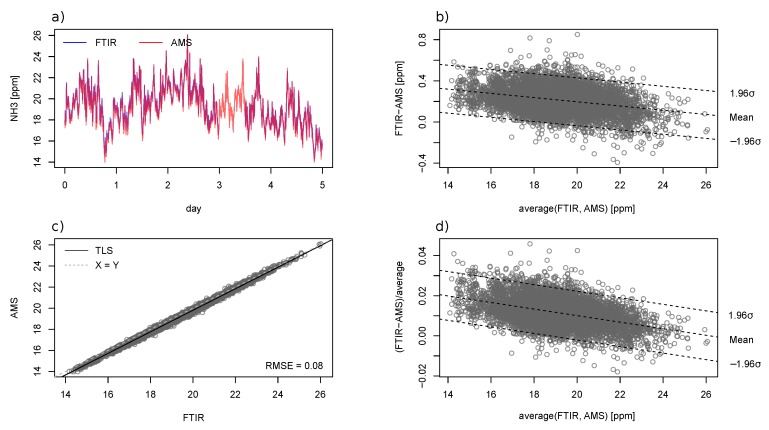
Comparison between the AMS and FTIR measurement system on NH_3_ measurement. The sub-plots are: the (**a**) time-series plot, (**b**) Bland–Altman plot of absolute differences, (**c**) scatter plot, and (**d**) Bland–Altman plot of relative difference. The dashed lines in the Bland–Altman plots represent the regression line and 95% bound of residuals.

A high agreement, R^2^ = 0.99, was found between the AMS and the FTIR in the empty compartment test (Figure 9), with RMSE_TLS_ = 0.08 ppm_v_ and rRMSE_TLS_ = 0.5%. In total, 6345 valid measurement points were obtained by FTIR at a 60-s update rate over the five-day test period. Approximately 12 h of data were lost due to FTIR software malfunctioning on Day 3. The measured indoor NH_3_ concentrations varied between 14 and 26 ppm_v_ during the five test days. Although no pigs were present during the time of the measurement, this concentration range was comparable to conditions when pigs were present. The indoor temperature of the compartment ranged between 2.4 and 15.3 °C. The pressure in the sampling line remained at 0.85 ± 0.01 bar absolute. The TLS regression line was given by AMS=−0.625+1.021·FTIR. Both the absolute (Figure 9b) and relative difference (Figure 9d) between the two analyzers were associated with the concentration level.

### 3.3. Discussion on Instrument Comparison

The results from the two field tests were generally satisfying. Based on the sensor specification and findings in earlier studies [23,24], a measurement error <2 ppm_v_ was expected from the AMS. This was in line with the observed accuracy in both tests. Temporal variations of NH_3_ concentration were well captured by both the AMS and FTIR. Similar Bland–Altman plots were obtained from the two field tests. Theoretically, instruments with an equivalent accuracy should satisfy slope coefficient = 1 and offset = 0. The linear dependency of the absolute difference (Figure 6b and Figure 9b) and relative difference (Figure 6d and Figure 9d) respectively suggested a bias in the calibration slope and offset [33].

The degree of agreement between the two systems on the measured NH_3_ concentration was clearly higher in the empty compartment. The experimental setup of the empty compartment test should be more appropriate for instrument comparison. Using a shared sampling tube ensured an identical composition of the supplied air samples for the AMS and the FTIR up to the point where the tube was split, and thus the two devices were expected to agree on the NH_3_ concentration. Nonetheless, there was still a time misalignment between the data series from the two systems due to differences in tube length between the split point and the analyzers, the air flow rate, and the system clock time. Cross-correlation was used in this study to find the relative time difference and align the data series in order to preserve the time resolution. Alternatively, averaged values over a wide time-frame, such as in [34], could be used for the comparison to suppress the effect of time misalignment at the cost of lower time resolution. In the occupied compartment test, because of the distinctive sampling process between the AMS and the FTIR, sampling errors could also contribute to the disagreement between instruments in addition to the sensor error and data misalignment. In the AMS sampling line, the particle filter was made of stainless steel. Due to a high NH_3_ adsorption rate [35], the material is less recommended for sampling NH_3_. It was reported in earlier studies that stainless steel would prolong the rise and fall time of a step response [35,36], and acquiring concentration values before convergence would lead to biased measurements. This may partially explain the larger variation in the difference between the AMS and FTIR during the occupied compartment test. Nonetheless, since adsorption is a reversible process, stainless steel is not expected to be the cause of the persistent over- or under-estimation observed in the occupied compartment test. To verify this speculation, a follow-up experiment was conducted where NH_3_ measurements were taken by the AMS over 12 days from two sampling tubes, equipped with a stainless-steel and a Teflon filter, respectively, placed at the same location in a pig compartment. Across the 10–50 ppm_v_ observed range, 95% of the momentary differences between the two sampling lines varied up to 4.5 ppm_v_ or 15.4%, but the daily average differences lied within −2.2–0.9%.

**Figure 10:**
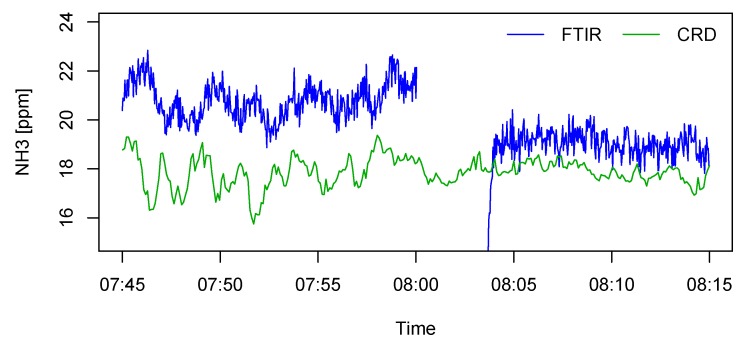
Offset shift of the Fourier transform infrared (FTIR) spectroscopy gas analyzer after zero calibration at 8:00. Comparative measurements were provided by a cavity ring-down (CRD) spectroscopy analyzer.

The offset of the LGD F200 could shift if the analyzer housing was enriched with NH_3_. The reason was that there was an air space inside the device between the laser diode and measurement chamber, and the presence of NH_3_ in this air space would also contribute to the sensor output, leading to an offset shift by up to 1 ppm_v_ according to the experience of the authors. The analyzer housing was purged with dry nitrogen at the beginning of the test, but purging could not prevent NH_3_ from entering the housing afterwards. In the case of this study, although the instrument room was isolated from the rearing area, the ambient NH_3_ level might elevate if the exhaust air from the analyzers leaked into the room. On the other hand, housing contamination would theoretically result in an overestimated concentration, so it could not explain the lower estimated value by the AMS compared to the FTIR. The FTIR was, in principle, also subjected to housing contamination, but to a much lesser extent. However, a subsequent test suggested that the offset of the FTIR could still undergo evident drifts. An example is given in Figure 10, where NH_3_ concentrations from a fattening pig compartment were measured by the FTIR and a cavity ring-down spectroscopy (CRD) analyzer (Model G2508, Picarro Inc., Santa Clara, CA, USA) using a shared sampling tube. In this example, the FTIR offset was shifted by −0.8 ppm_v_ after performing a zero calibration. The magnitude of the shift was comparable to the mean difference between the AMS and the FTIR during the occupied compartment test.

### 3.4. Practical Perspectives and Further Development

Performance-wise, the results in this study showed that the AMS was capable of maintaining adequate accuracy over five days, and for longer periods, the potential drift was within 1 ppm_v_, which would be sufficient for general research purposes in pig houses. Moreover, the data acquisition with the AMS could be performed automatically in real time, and this provides a directly practical convenience compared to manual methods such as wet chemistry.

In this study, the temperature of the ambient and air samples was maintained at the same level, but air conditioning may not be available under other field conditions. Temperature could potentially affect the spectra line shape of gas molecules [32] and thereby reduce the accuracy of AMS. Humidity and matrices of the air samples may also influence the measurement accuracy due to cross-sensitivity. To further ensure and improve the reliability, it is important to verify if the AMS could still measure accurately at varying sample temperature, humidity, and compositions.

The pressure dependency of the LGD F200 could be an issue for certain sampling methods. For example, the Verification of Environmental Technologies for Agricultural Production (VERA) protocol suggests using sampling lines equipped with critical orifices [6], and to reach a critical flow, the inline pressure needs to be lower than 0.528× ambient pressure [37] if a vacuum pump is used. At such low pressure, the LGD F200 output would be significantly biased. A pressure compensation solution was proposed in this study, but the data obtained in this study were unsuited for assessing the accuracy of the compensation equation in field conditions due to either a lack of pressure variation or the involvement of other uncertainties.

The cost of the AMS (excluding the CO_2_ module) was approximately €9500, which is considerably more affordable than other commercially-available high-end gas analyzers. In addition to using a relatively low-cost analyzer module, the cost aspect was taken into account in the system design and selection of components. For example, an alternative but potentially more costly solution to the pressure dependency would be using a compressor pump in the upstream. Because of the physiochemical property of NH_3_, the wetted part of the compressor pump had to be not only chemically resistant, but also of low absorptivity. Using an Arduino-compatible microcontroller also allowed keeping the cost low. In addition to serving as a logger, the microcontroller would also be used for controlling a multi-sampler in the further development. By enabling the AMS to measure multiple locations, the average measurement cost would be reduced with an increasing number of sampling locations.

## 4. Conclusions

In this study, a cost-effective ammonia (NH_3_) monitoring system (AMS), which incorporated a tunable diode laser spectroscopy analyzer module, was constructed and evaluated in both laboratory and field conditions. A good agreement between the AMS and a high-end Fourier transform infrared (FTIR) spectroscopy analyzer was found under two field conditions. The R^2^ between the two systems was respectively 0.64 and 0.99, with 0.66 ppm_v_ and 0.08 ppm_v_ RMSE_TLS_ and 5.9% and 0.5% rRMSE_TLS_. Possible sources of error in both the AMS and the FTIR measurements were discussed. The AMS was capable of performing continuous NH_3_ measurement and provided real-time output with comparable accuracy to FTIR, but the cost of the system was considerably lower than the high-end gas sensing instruments. Therefore, the AMS can be regarded as a promising cost-effective tool for quantifying emission factors in mechanically-ventilated animal houses and studies on emission processes in pig houses.

## Figures and Tables

**Table 1:** Valve positions in the laboratory pressure test setup using a gas cylinder.

Test	Pressure Regulator	Needle Valve			
		1	2	3	4
Super-atmospheric	Adjusted	Fully open	Fully closed	Adjusted	Fully open
Sub-atmospheric	Fixed	Adjusted	Fully open	Fully open	Adjusted
Leak	Fully closed	Fully open	Fully closed	Fully open	Fully closed

## Abbreviations

The following abbreviations are used in this manuscript:

AMS	Ammonia monitoring system
CRD	Cavity ring-down
FTIR	Fourier transform infrared
I.D.	Inner diameter
PTFE	Polytetrafluoroethylene
RMSE	Root-mean-squared-error
rRMSE	Relative-root-mean-squared-error
TDLA	Tunable diode laser absorption
TLS	Total least squares
